# High levels of contact dermatitis and decreased mobility in broiler breeders, but neither have a relationship with floor eggs

**DOI:** 10.1016/j.psj.2020.04.010

**Published:** 2020-04-24

**Authors:** Anna C.M. van den Oever, J. Elizabeth Bolhuis, Lotte J.F. van de Ven, Bas Kemp, T. Bas Rodenburg

**Affiliations:** ∗ Vencomatic Group, 5520 AD Eersel, The Netherlands; † Adaptation Physiology Group, Wageningen University, 6700 AH Wageningen, The Netherlands; ‡ Animals in Science and Society, Faculty of Veterinary Medicine, Utrecht University, 3508 TD Utrecht, The Netherlands

**Keywords:** broiler breeder, genetic line, leg health, foot pad dermatitis, floor egg

## Abstract

Contact dermatitis, both on the foot pads and hocks, is a well-known health issue in broilers. Less is known about contact dermatitis in broiler breeders, however, although they have many risk factors for developing leg health problems in common with broilers. This study aimed to describe the prevalence and severity of contact dermatitis during the production cycle in 5 lines of broiler breeders, investigate possible causes of contact dermatitis, and study its relationship with gait, egg production, and floor egg percentage. Five commercially available genetic lines of broiler breeders were housed in 21 pens of 550 females and 50 males from 20 to 60 wk of age. Every 10 wk litter quality, leg health measurements (foot pad dermatitis, hock burn, and gait) and body weight were assessed of 50 random hens per pen. Total number of eggs, number of eggs laid outside the nest (floor eggs), and mortality were recorded daily per pen. Prevalence of foot pad dermatitis, hock burn, and gait problems increased with age. Litter quality started to decrease at 50 wk of age. Prevalence of foot pad dermatitis was affected by litter quality, whereas genetic line had little effect. One genetic line was more prone to developing hock burns, though generally the prevalence of hock burn (13%) was much lower than that of foot pad dermatitis (74%). The percentage of broiler breeders with gait problems increased up to 24% with age, but this was not related to the prevalence of contact dermatitis. The lines differed in body weight from 32 wk of age onwards, and a higher body weight was related to lower egg production and higher cumulative mortality. The percentage of floor eggs was not related to leg health parameters or genetic line. Broiler breeders thus have similar leg health problems as broilers, but these problems are not related to the percentage of floor eggs, suggesting that other factors are involved in the undesirable behavior of floor laying.

## Introduction

Foot pad dermatitis and hock burns in broilers pose a major welfare problem and have received much attention over the last decades, but little is known on these conditions in broiler breeders. Both conditions are a form of contact dermatitis, where the skin becomes acutely inflamed when it comes into contact with irritating material (Greene et al., 1985). The incidence of foot pad dermatitis in fast-growing broiler flocks has been found to be up to 65% at slaughter age, whereas the incidence of severe hock burn at this age can be as high as 41% (Haslam et al., 2007, de Jong et al., 2012, Bassler et al., 2013). Limited research has been done on the prevalence of contact dermatitis in broiler breeders, but it seems that this condition is also prevalent in the parent stock (Wolanski et al., 2004, Renema et al., 2007, Kaukonen et al., 2016).

The risk factors for developing contact dermatitis that have been identified in broilers are present in the broiler breeder husbandry as well. Poor litter quality, where a high litter moisture level causes more ammonia release, is the most common risk factor found for foot pad dermatitis (Martland, 1985). Broiler breeder flocks are kept over 6 times longer in the production house than broilers, and during this period, very little fresh litter is usually added, which results in accumulating litter (manure) and increasing litter moisture (Lien et al., 1998, Maguire et al., 2006). However, broiler breeder farms usually have slatted areas, which give the birds an opportunity to rest in a nonlitter area, and this might have beneficial effects on contact dermatitis (Sander et al., 2003). Genetic predisposition and high body weight are other risk factors in broilers (Shepherd and Fairchild, 2010) and may also play a role in the development of contact dermatitis in broiler breeders.

Contact dermatitis is likely a painful condition, which is associated with health and performance problems (Ekstrand et al., 1998, De Jong and Guémené, 2011). Lame broilers were found to consume more feed containing analgesic drugs than unaffected broilers, and contact dermatitis is one of the conditions that can cause lameness (Danbury et al., 2000). Broilers have been found to have a decreased feed intake and a higher chance of infection with microorganisms such as *Staphylococcus aureus* with increasing foot pad dermatitis (Martland, 1985, Hester, 1994). In broiler breeders, painful contact dermatitis could reduce the hen's ability of reaching the nest and lead to an increased risk of eggs laid outside the nest, so-called floor eggs. Floor eggs are an economic problem as they require manual collection and are often dirty, which results in a lower saleability and hatchability (van den Brand et al., 2016). Floor eggs may also reflect a welfare problem as chickens are inclined to lay their egg in a secluded nest (Zupan et al., 2008, Buchwalder and Fröhlich, 2011), which suggests that a hen laying her egg elsewhere is constrained by, for example, reduced mobility because of contact dermatitis.

To our knowledge, little is known about the prevalence of leg health issues and their consequences for production and welfare in broiler breeders. The first aim of this study was therefore to assess the prevalence and severity of contact dermatitis in broiler breeders during the production cycle. Furthermore, we investigated possible risk factors associated with contact dermatitis by comparing different commercially available genetic lines, measuring body weight, and scoring for gait and litter quality. Finally, the relationships between contact dermatitis, gait, egg production, and floor egg percentage were investigated. We expected an increase in prevalence and severity of both foot pad dermatitis and hock burns with age. Genetics, body weight, and litter quality were expected to have an effect on both forms of contact dermatitis. Foot pad dermatitis was expected to be related to increased gait problems, which in turn was predicted to be related to a higher percentage of floor eggs.

## Materials and methods

### Animals and Housing

The experiment took place from June 2018 to March 2019 at a breeding station. A total of 11,550 females and 1,050 males, all non-beak trimmed, were moved from their rearing facilities located at the same breeding station at the age of 20 wk. Five commercially available fast-growing genetic lines were represented in different numbers. The chickens were assigned to 21 pens of 550 females and 50 males of the same genetic line, resulting in 6 pens for lines 1 and 2 (3,300 females and 300 males per line), 5 pens for line 3 (2,750 females and 250 males), and 2 pens for lines 4 and 5 (1,100 females and 100 males per line). The position of the genetic lines in the house was randomized using a block design. The pens were identical in size (12 × 6.5 × 2.0 m, length × width × height) and lay-out and were placed in 4 rows. Animal density was 7.7 birds/m^2^, which is comparable to commercial practice. The pens had wire mesh walls, which allowed the animals from different pens to see each other. The litter area (12 × 3.7 m) was covered with wood shavings, and the slatted area (12 × 2.3 m) was raised by 0.5 m and gave access to 9 bell drinkers and 10 nests. No wood shavings were added, removed, or otherwise treated during the duration of the experiment. The group-nests were of a rollaway type (Vencomatic), measuring 1.15 × 0.52 × 0.53 m (l × w × h). All nests had a green rubber nest floor slanting toward the back and red nest curtains with an entry in the middle. The feeding line for the females was placed partially on the slats and partially in the litter area, whereas the male feeding line was positioned in the litter area.

The house was lit with artificial LED-lighting. At 20 wk of age, the animals had 8 h of light (7:00–15:00 h) at 10 lx measured at bird height. This was gradually increased with age and laying percentage of the flock to 14 h of light (2:00–16:00 h) at 60 lx at bird height. The temperature was maintained at 21°C ± 1°C, using a combination of air intake ventilators, exhaust valves, and a heat exchanger. Food was provided at 8:30 h, giving a restricted amount according to the management guide of the breeding companies ranging from 100 to 164 g. Birds were continuously weighed automatically with a poultry scale hanging in the litter area. Water was provided from 8:30 to 12:30 h and from 15:30 to 16:00 h. The nests were available to the hens from 1 h before lights-on until 30 min before lights-off, from the day after the first egg was found (23 wk of age). The birds were kept until the age of 60 wk and then slaughtered for human consumption.

### Data Collection

#### Leg Health

**Figure 1:**
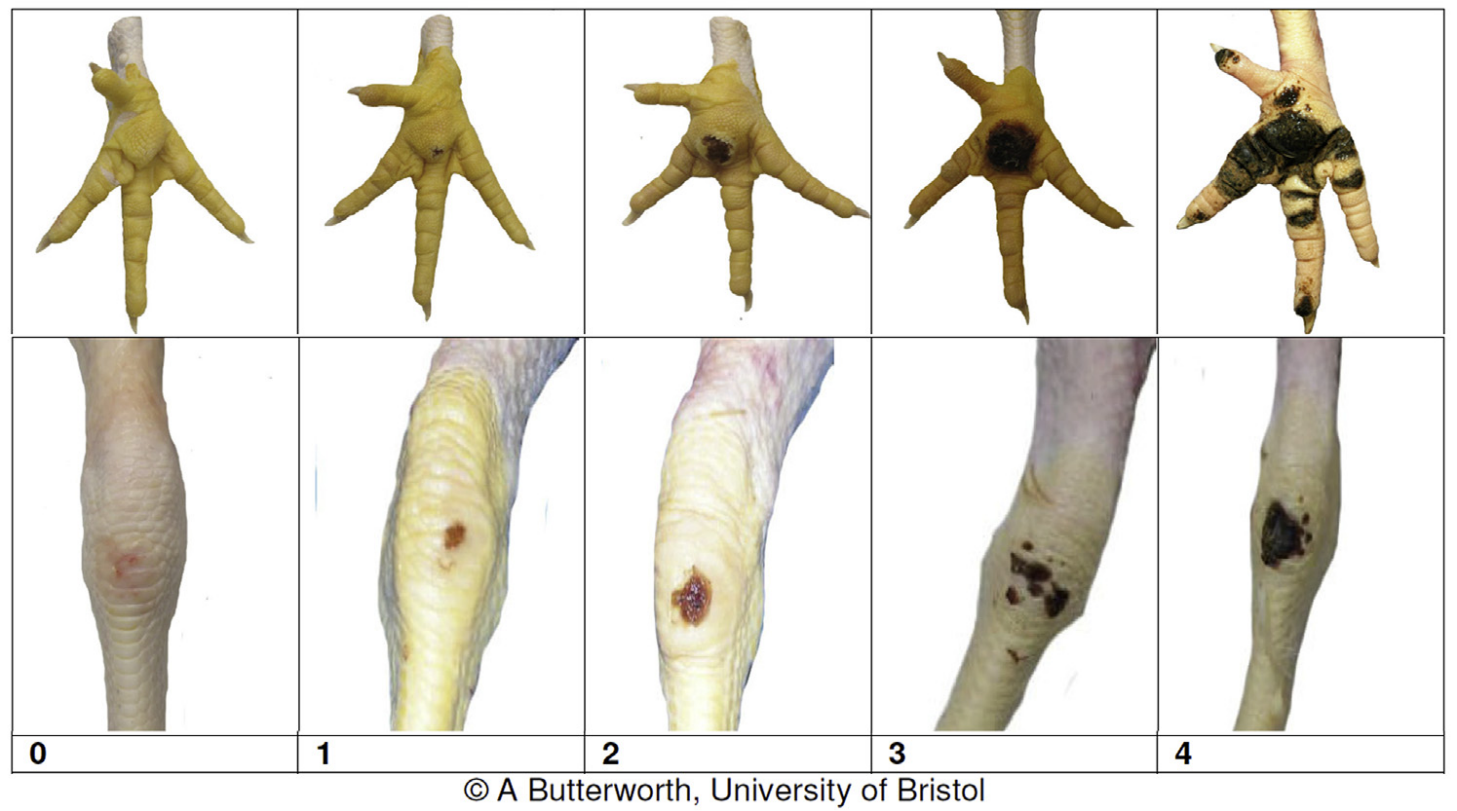
Visual scales for scoring foot pad dermatitis and hock burn (Welfare Quality, 2009).

Approximately every 10 wk (21, 32, 40, 50, and 60 wk of age), a random selection of 50 hens per pen was hand-weighed and scored for foot pad dermatitis and hock burn according to the Welfare Quality Assessment Protocol for Poultry (2009), see Figure 1. A score 0 represents no dermatitis, 1–2 mild dermatitis, and 3–4 severe dermatitis. Litter quality was recorded according to the score described in the Welfare Quality Assessment Protocol for Poultry (2009), ranging from 0 for dry and flaky litter to 4 for solid litter covered with a crust. Gait was scored according to a scale adapted from Garner et al. (2002), see Table 1. A score 0 represents no gait problems, 1–2 mild gait problems, and 3–5 severe gait problems. Any mortalities were noted.

**Table 1:** Scale for scoring gait, adapted from Garner et al. (2002).

Gait score	Description
0	Bird moves fluidly.
1	Bird has an unsteady, wobbling walk. Problem leg cannot be detected.
2	Bird walks for more than 10 s. Problem leg can be detected.
3	Bird walks away spontaneously but squats within 10 s.
4	Bird only walks away when approached or nudged.
5	Bird cannot walk.

#### Production

Starting at 24 wk of age until the birds were depopulated, the number of floor and nest eggs were recorded daily per pen. Floor eggs were collected 3 times per day, and nest eggs were collected once a day. Eggs laid on the slatted area were prevented from rolling into the litter with a 18 mm plastic tube, which allowed for separate recording of litter eggs and eggs laid on the slats. This plastic tube had to be removed at 45 wk of age for manure management, after which no distinction could be made between floor eggs laid on the slats or in the litter.

This study was not considered to be an animal experiment under the Law on Animal Experiments, as confirmed by the local Animal Welfare Body (3 June 2018, Wageningen, The Netherlands).

### Statistical Analysis

Egg production percentage per pen was calculated by dividing the total number of eggs by the number of hens present. Floor egg percentage was calculated by dividing the number of floor eggs over the total number of eggs laid per pen per week, whereas litter egg percentage was calculated by dividing the number of eggs laid in the litter over the total number of floor eggs. Mortality percentage per pen was calculated by dividing the number of dead hens over total number of hens placed. All leg health parameters were analyzed as mean per pen per observation week. The percentage of birds was calculated for presence of foot pad dermatitis, hock burn, and gait problems (score ≥ 1) as well as for severe foot pad dermatitis, hock burn, and gait problems (scores ≥ 3).

All statistical analyses were performed with SAS (version 9.4). The MIXED procedure was used to perform repeated general linear mixed models to investigate differences between lines and weeks of age. Fixed effects included line and week of age and their interaction, and pen within line was included as a random effect. The assumptions of homogeneity of variance and normally distributed residuals were examined visually using the conditional studentized residuals plots. Pearson correlations were calculated between traits using the CORR procedure. Results are shown as nontransformed means with corresponding standard errors, and *P*-values below 0.05 were considered significant. Tukey's post hoc test was performed to investigate significant differences between test groups.

## Results

### Leg Health

**Figure 2:**
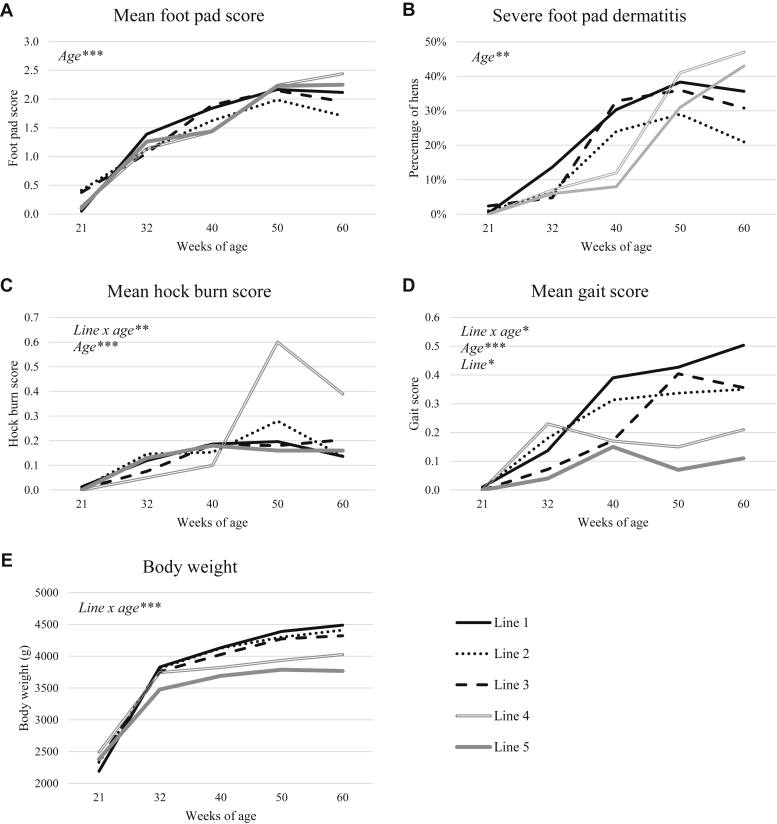
Overview of the development of leg health parameters and body weight with age, specified per genetic line. (A) The mean foot pad dermatitis score. (B) The percentage of hens with a severe foot pad dermatitis score of 3–4. (C) The mean hock burn score. (D) The mean gait score. (E) The mean body weight in grams. Significant effects of line, age, or the interaction between line and age are noted in italic in the top left corner with an indication of the *P*-value (∗<0.05; ∗∗ < 0.01; ∗∗∗ < 0.001). Scores of 0 indicate unaffected birds, whereas scores of 3 and higher are considered severe.

For an overview of the results of all measured leg health indicators specified per genetic line, see Figure 2. For an overview of leg health indicators, body weight and litter quality over age, see Table 2.

**Table 2:** Mean values and standard errors of leg health variables measured, specified per age. Severe foot pad score, hock burns, and gait problems indicate the percentage of hens affected. Scores of 0 indicate unaffected birds, while scores of 3 and higher are considered severe.

Item	21 wk	32 wk	40 wk	50 wk	60 wk
Mean foot pad score	0.3 ± 0.0^d^	1.2 ± 0.1^c^	1.7 ± 0.1^b^	2.1 ± 0.1^a^	2.0 ± 0.1^a^
Foot pad score ≥1 [% of birds]	17.5 ± 1.2^d^	77.5 ± 1.3^c^	85.1 ± 1.1^b^	95.0 ± 0.7^a^	93.0 ± 0.8^a^
Foot pad score 3–4 [% of birds]	1.0 ± 0.3^d^	8.1 ± 0.8^c^	25.2 ± 1.3^b^	34.7 ± 1.5^a^	32.1 ± 1.4^a^
Mean hock burn score	0.0 ± 0.0^d^	0.1 ± 0.0^c^	0.2 ± 0.0^b,c^	0.3 ± 0.0^a^	0.2 ± 0.0^a,b^
Hock burn score ≥ 1 [% of birds]	0.7 ± 0.3^d^	10.8 ± 1.0^c^	15.4 ± 1.1^b^	20.9 ± 1.3^a^	16.0 ± 1.1^b^
Hock burn score 3–4 [% of birds]	0.0 ± 0.0^a^	0.0 ± 0.0^a^	0.3 ± 0.2^a^	0.2 ± 0.1^a^	0.4 ± 0.2^a^
Mean gait score	0.0 ± 0.0^c^	0.1 ± 0.0^b^	0.3 ± 0.0^a^	0.3 ± 0.0^a^	0.4 ± 0.0^a^
Gait score ≥ 1 [% of birds]	0.4 ± 0.2^d^	8.7 ± 0.9^c^	18.8 ± 1.2^b^	24.4 ± 1.3^a^	23.3 ± 1.3^a,b^
Gait score 3-5 [% of birds]	0.0 ± 0.0^b^	1.0 ± 0.3^a,b^	2.3 ± 0.5^a^	1.0 ± 0.3^a,b^	2.7 ± 0.5^a^
Body weight [g]	2,319 ± 12^e^	3,766 ± 10^d^	4,035 ± 12^c^	4,236 ± 15^b^	4,314 ± 16^a^
Litter quality	0.0 ± 0.0^c^	0.0 ± 0.0^c^	0.3 ± 0.1^c^	1.6 ± 0.1^b^	3.2 ± 0.2^a^

^a-e^Means lacking a common superscript within a row differ (*P* < 0.05).

The condition of foot pads significantly deteriorated as the hens aged, illustrated by higher scores, until it stabilized at the age of 50 and 60 wk (F_4,64_ = 127.65, *P* < 0.0001). Foot pad condition was not affected by genetic line (F_4,16_ = 0.24, *P* = 0.9) or its interaction with age (F_16,64_ = 1.62, *P* = 0.08). In line with the increasing foot pad lesion scores, the percentage of hens with severe foot pad dermatitis (score 3 or 4) increased from 32 to 50 wk age, after which it stabilized (F_4,64_ = 34.63, *P* < 0.001), with no significant differences between the lines (F_4,16_ = 0.70, *P* = 0.6) and no line by age interaction (F_16,64_ = 1.41, *P* = 0.2). Average foot pad dermatitis score per pen correlated with litter quality scores (r = 0.47, *P* = 0.034), with most problems in pens with the poorest litter. Foot pad score was not significantly correlated with body weight at the individual level.

The mean hock burn score was affected by the interaction between age and line (F_16,64_ = 3.00, *P* = 0.0009) and the main effect of age (F_4,64_ = 23.02, *P* < 0.0001), but not by the main effect of line (F_4,16_ = 1.65, *P* = 0.2). Only at 50 and 60 wk of age, line 4 had a higher score (0.6 ± 0.1 and 0.4 ± 0.1 respectively) than the other lines (0.1–0.3 and 0.1–0.2, respectively), whereas the lines did not differ at the earlier ages. The mean hock burn score increased until the age of 50 wks after which it stabilized. The low mean hock burn score is reflecting of the low severity of hock burn, maximum 0.4 ± 0.2% of the birds received a score of 3, whereas none received a score of 4. Hock burn score was not significantly correlated with body weight at the individual level or with litter quality score at pen level.

Mean gait score was affected by the interaction between age and line (F_16,64_ = 2.2, *P* = 0.014) and the main effect of age (F_4,64_ = 22.2, *P* < 0.0001) as well as genetic line (F_4,16_ = 4.29, *P* = 0.02). Line 1 had a higher gait score than line 3 at the age of 40 wk (0.4 ± 0.0 compared with 0.2 ± 0.0), and line 1 had a higher gait score than line 5 at 60 wk of age (0.5 ± 0.1 compared with 0.1 ± 0.0). All other lines had intermediate scores. Independent of line, the mean gait score increased to 0.3 ± 0.0 at the age of 40 wk, after which it stabilized. When looking at the overall differences between lines, line 1 (0.29 ± 0.02) had a higher mean gait score than line 5 (0.07 ± 0.01), and all other lines had intermediate scores. The percentage of birds with severe gait scores (scores 3-5) increased with age (F_4,64_ = 3.7, *P* = 0.01), but was not affected by genetic line (F_4,16_ = 1.5, *P* = 0.3) or its interaction with age (F_16,64_ = 1.4, *P* = 0.2). At 40 and 60 wks of age, the highest percentages of 2.3 ± 0.5% and 2.7 ± 0.5%, respectively, were observed. Gait score was not significantly correlated with foot pad score, hock burn score, or body weight within individuals.

Body weight was affected by the interaction between line and age (F_16,64_ = 9.34, *P* < 0.0001). The differences between the lines started at the age of 32 wk, when line 1 (3,832 ± 19 g) had a significantly higher body weight than line 5 (3,478 ± 27 g) with line 2 (3,810 ± 20 g), line 3 (3,758 ± 19 g), and line 4 (3,745 ± 32 g) in between. At 40 wks of age, both line 1 and 2 (4,133 ± 24 g and 4,126 ± 22 g respectively) were significantly heavier than line 5 (3,692 ± 33 g), whereas at 50 wks of age, lines 1-3 (4,391 ± 27 g, 4,301 ± 27 g and 4,373 ± 28 g respectively) were significantly heavier than line 4 and 5 (3,934 ± 38 g and 3,789 ± 30 g respectively). At the age of 60 wk, lines 1-3 (4,489 ± 30 g, 4,411 ± 27 g and 4,323 ± 32 g respectively) had a significantly higher body weight than line 5 (3,768 ± 36 g), whereas line 4 had an intermediate body weight (4,026 ± 42 g).

The cumulative mortality differed between the lines (F_4,16_ = 8.65, *P* = 0.0006). Line 1 had a higher mortality (10.3 ± 1.9%) than lines 2 (6.9 ± 0.4%), 3 (4.8 ± 0.4%), and 5 (4.3 ± 0.9%), whereas line 4 had an intermediate level of mortality (7.5 ± 0.2%). Cumulative mortality was positively correlated with average body weight per pen (r = 0.43, *P* = 0.052), with higher mortality in pens with heavier birds.

Litter quality deteriorated with the age of the birds (F_4,64_ = 173.96, *P* < 0.0001) but was not affected by line (F_4,16_ = 1.54, *P* = 0.2) or the interaction between age and line (F_16,64_ = 1.56, *P* = 0.1). The mean litter score increased significantly from 50 wk of age and 60 wk of age compared with 21, 32, and 40 wk of age.

### Production

**Figure 3:**
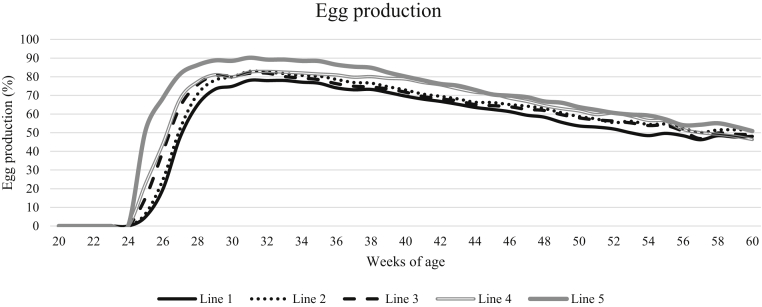
The development of egg production percentage with age, specified for the different genetic lines.

Egg production percentage increased rapidly after the onset of lay and then declined again after the age of 30 wk, see Figure 3. An interaction between line and age was found, where lines differed at earlier ages but not at the age of 60 wk (F_140,5000_ = 23.66, *P* < 0.0001). Line 5 had a significantly higher egg production percentage than line 1 from the onset of lay up to 50 wk of age, whereas line 4 only had a significantly higher egg production percentage than line 1 during ages 40 to 50 wk. Egg production percentage was negatively correlated with average body weight per pen (r = −0.50, *P* = 0.022).

**Figure 4:**
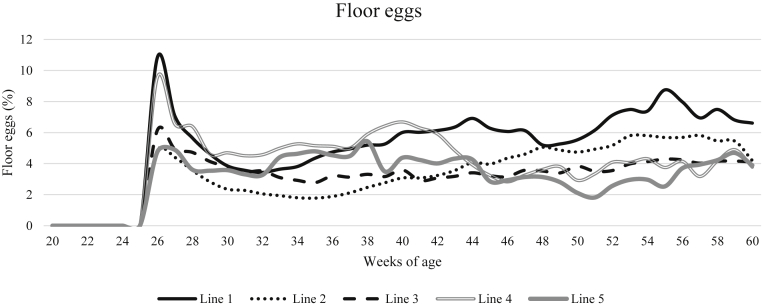
The development of percentage of floor eggs with age, specified for the different genetic lines.

The percentage of floor eggs was affected by the interaction between line and age (F_136,4849_ = 11.28, *P* < 0.0001), see Figure 4. The percentage of floor eggs of all genetic lines fluctuated with age, and no clear pattern could be distinguished in this interaction. Most of the floor eggs were found in the litter area. The percentage of litter eggs (expressed as a percentage of floor eggs) increased significantly from 78.3 ± 2.0% at 26 wk of age to 88.9 ± 1.1% at 30 wk of age (F_20,2840_ = 15.0, *P* < 0.0001) after which it did not increase anymore. The lines did not significantly differ in percentage of litter eggs (F_4,16_ = 0.97, *P* = 0.4). No significant correlations between floor egg percentage and leg health parameters or body weight were found.

## Discussion

This study aimed to provide descriptive information on the prevalence and severity of contact dermatitis in broiler breeders during the production cycle, which we found to increase with age. Hock burn was less prevalent and less severe than foot pad dermatitis, both of which were not correlated to body weight. Body weight did correlate to egg production, which differed significantly between the commercially available genetic lines. The percentage of floor eggs was, against our hypothesis, not correlated to contact dermatitis or gait problems.

### Leg Health

In accordance with our expectations, the incidence and severity of all indicators for leg health problems increased with age. Our expectations were mainly based on broiler research, where the prevalence of severe foot pad dermatitis in commercial flocks ranges from 38 to 72% at slaughter age (Haslam et al., 2007, de Jong et al., 2012). Only 1 study has been published describing leg health during the production cycle of broiler breeders, which found 0 to 5.5% of birds had severe foot pad dermatitis at 19, 24, and 36 wk of age, after which it significantly increased to 25% at the age of 48 wks and increased further toward 64% at 60 wk of age (Kaukonen et al., 2016). This is largely in accordance with our findings, although we did not find an increased percentage of birds with severe foot pad dermatitis between 50 and 60 wk of age (35% and 32%, respectively). Kaukonen et al. (2016) stressed the large variation between flocks of severe foot pad dermatitis prevalence at slaughter age, ranging from 51 to 83% in their study. This could explain the difference between our findings and the study of Kaukonen et al. at later ages. Our study was performed with large groups housed under commercial, but controlled, conditions. This allowed us to gather information that reflects the commercial industry without the large variety in management and environmental factors of a field study. Both our study and that of Kaukonen et al. (2016) report that at least 30% of broiler breeder hens have severe foot pad dermatitis at a certain point in the production cycle, indicating that this is an important problem in the breeder industry.

The severity of foot pad dermatitis was correlated with decreasing litter quality from the age of 40 wk onward. This relation between foot pad dermatitis and litter quality has been well established in broiler research, as well as in the previously mentioned studies with broiler breeders (Shepherd and Fairchild, 2010, Kaukonen et al., 2016). The moisture of litter is the most important factor in the development of foot pad dermatitis (Martland, 1985). The scoring system for litter quality used in this study is designed to reflect the level of moisture in the litter. However, Kaukonen et al. (2016) used the same scoring system and found that litter with a higher score had a lower moisture content. Although litter moisture content was not measured in this study, the litter appeared more moist with a higher litter score.

The higher incidence of hock burn in line 4 at later ages was not related to deteriorating litter quality or gait, which suggests that this line has a genetic predisposition for hock burn development. Earlier studies with broilers have also found differences between genetic strains for the prevalence of hock burns (Kestin et al., 1999, Haslam et al., 2007, Ask, 2010). Lines 4 and 5 were represented with a lower amount of birds than lines 1-3 because of constraints in the experimental set-up, so these results should be carefully interpreted, and more research is needed on the genetic influence on contact dermatitis.

The severity of hock burns was very low in this experiment with 0.4% of the birds having severe hock burn at the age of 60 wk. Even so, this is a higher severity compared with the research of Kaukonen et al. (2016), who did not find any broiler breeders with severe hock burn. This could be because of differences in genetic line, stocking density, or litter quality compared with our study. However, the percentage of birds with severe hock burn in our study is still much lower than the average percentage of broilers found to have severe hock burn before slaughter. Findings range from an average of 1.3 to 7.9%, with some farms having more than 40% of the birds affected with severe hock burn (Haslam et al., 2007, Bassler et al., 2013).

Hock burns in broilers have been found to correlate with body weight, as it is thought that the heavier broilers spend more time sitting. This increased amount of time of hocks spent in contact with the litter increases the incidence of hock burn (Kjaer et al., 2006, Haslam et al., 2007). We did not find a correlation between hock burn and body weight, however. It is possible that broiler breeders do not alter their sitting behavior with increasing body weight as they grow more gradually than broilers. Another explanation would be that the birds mostly use the slatted areas to rest on and decrease the contact with litter in this manner. Our study did not include any activity measurements to validate this suggestion.

The incidence of gait problems increased with age and differed between the genetic lines, which was also found in another study with different genetic lines of broilers (Kestin et al., 1999). The severity of gait problems was low in our experiment with a maximum of 2.7% of the hens having severe gait problems. No other studies with broiler breeders are available for comparison, but our results can be compared with studies on commercial broilers. Two studies found that on average 14 to 30% of the broilers showed gait problems with some flocks having 50% of the birds affected (Sanotra et al., 2003, Bassler et al., 2013). Against expectations, we did not find correlations between foot pad dermatitis and gait score, so it seems that (also) other factors are at the basis of gait problems, such as activity of the birds. Walking ability has been shown to improve by increasing the activity of broilers, as this enhances tibiotarsal bone thickness and decreases vasculature abnormalities of bone extremities (Sherlock et al., 2010). This is likely also the case for broiler breeders, although future studies are necessary to confirm this suggestion.

Although all lines were given the same amount of feed, their body weights differed significantly from 32 wk of age. This suggests a difference in feed conversion ratio that has a genetic basis because of selective breeding for desired traits at offspring level (Dawkins and Layton, 2012). A higher body weight has mainly negative consequences for the health of the birds. This is illustrated by the positive correlation between body weight and cumulative mortality in our study. It should, however, be noted that it is also possible that a higher mortality allowed the remaining birds to grow faster because of increased (feeding) space.

### Production

Egg production percentage followed a pattern as commonly seen in commercial practice (Zuidhof et al., 2007), although the genetic lines differed significantly. After an initial steep increase until the age of 30 wk with a mean egg production of 83%, the percentage slowly decreased again to 50% at the slaughter age of 60 wk. Clear differences between the genetic lines were visible at all ages, except 60 wk of age. Differences in egg production percentage between genetic strains is a well-known phenomenon, because this is directly related to balancing different breeding goals for broilers in terms of growth and body weight (Dawkins and Layton, 2012).

While egg production percentage can thus be partially explained by genetic potential, we also found a negative correlation with body weight until the age of 50 wk. It has been well established that broiler breeders have to be fed restrictively to maximize egg production (Decuypere et al., 2010). The effect of body weight on reproductive traits has mainly been studied by comparing ab libitum and restrictively fed broiler breeder hens. Ad libitum fed hens have a lower egg production percentage than restrictively fed hens, mainly because of an increased number of defective eggs (for a review see Robinson et al., 1993). Furthermore, ad libitum fed hens were found to have a shorter laying sequence length than restrictively fed hens, which is indicative for erratic oviposition (Renema and Robinson, 2005). The relation between body weight and egg production could also be explained in the opposite direction, as a lower energy expenditure in egg production leaves more energy allocation for growth (Robinson et al., 1993).

The percentage of floor eggs did not differ between the genetic lines, which suggests that this is not a behavior directly affected by genetic selection but rather the outcome of a combination of other factors. Although the severity of foot pad dermatitis and the prevalence of gait problems increased with age, no correlation was found between either contact dermatitis or gait and the percentage of floor eggs. This suggests that decreased mobility is not the only factor involved in floor laying, and further research could focus on other possible explanations for floor laying behavior, such as the influence of social behavior and general activity levels. These relationships were studied in the same birds and are reported elsewhere (van den Oever et al., unpublished data).

In conclusion, this study shows that deteriorated leg health is an issue of similar size within the broiler breeder industry as it is in the broiler industry and should therefore receive more attention. It is confirmed that litter quality is related to the severity of foot pad dermatitis, whereas body weight only seems to be related to egg production and not to contact dermatitis. The genetic lines differ in some of the parameters measured. The percentage of floor eggs could not be attributed to genetic line, foot pad dermatitis, hock burn, or gait, which means that also other factors are involved in the development of the undesirable floor laying behavior.
